# Angiopep-2-Modified Nanoparticles for Brain-Directed Delivery of Therapeutics: A Review

**DOI:** 10.3390/polym14040712

**Published:** 2022-02-12

**Authors:** Saffiya Habib, Moganavelli Singh

**Affiliations:** Nano-Gene and Drug Delivery Group, Discipline of Biochemistry, School of Life Sciences, University of KwaZulu-Natal, Private Bag X54001, Durban 4000, South Africa; saffiya.habib@gmail.com

**Keywords:** Angiopep-2, nanoparticles, transcytosis, drug delivery, brain, targeting

## Abstract

Nanotechnology has opened up a world of possibilities for the treatment of brain disorders. Nanosystems can be designed to encapsulate, carry, and deliver a variety of therapeutic agents, including drugs and nucleic acids. Nanoparticles may also be formulated to contain photosensitizers or, on their own, serve as photothermal conversion agents for phototherapy. Furthermore, nano-delivery agents can enhance the efficacy of contrast agents for improved brain imaging and diagnostics. However, effective nano-delivery to the brain is seriously hampered by the formidable blood–brain barrier (BBB). Advances in understanding natural transport routes across the BBB have led to receptor-mediated transcytosis being exploited as a possible means of nanoparticle uptake. In this regard, the oligopeptide Angiopep-2, which has high BBB transcytosis capacity, has been utilized as a targeting ligand. Various organic and inorganic nanostructures have been functionalized with Angiopep-2 to direct therapeutic and diagnostic agents to the brain. Not only have these shown great promise in the treatment and diagnosis of brain cancer but they have also been investigated for the treatment of brain injury, stroke, epilepsy, Parkinson’s disease, and Alzheimer’s disease. This review focuses on studies conducted from 2010 to 2021 with Angiopep-2-modified nanoparticles aimed at the treatment and diagnosis of brain disorders.

## 1. Introduction

The blood–brain barrier (BBB) is a selectively permeable network of capillary endothelial cells, astroglia, pericytes, and perivascular mast cells, which stringently regulates the exchange of molecules between the blood and the cerebral tissue. The system functions in protecting the central nervous system (CNS), providing nutrients to the brain, maintaining homeostasis, and regulating communication to and from the CNS [1]. The former protective capability is conferred by the presence of tight intercellular junctions that prevent the entry of pathogens and toxins [2]. 

In line with the exclusion of foreign substances, the BBB is also a significant impediment to the delivery of therapeutic and diagnostic agents to the brain. Moreover, ATP-binding cassette (ABC) transporters of BBB endothelial cells can expel compounds that may traverse the barrier back into the bloodstream [3]. Consequently, brain disorders are notoriously difficult to diagnose and treat both by conventional methods and nanotechnology.

Understanding the natural routes of transport, such as receptor-mediated transcytosis (RMT), across the BBB has led to the ‘trojan horse’ concept being widely investigated. This strategy involves modifying nanoparticles (NPs) with ligands that can bind specific receptors at the apical membrane of brain endothelial cells and promote endocytosis. In this way, the entry of the NP is masked through recognition of the ligand. Angiopep-2 is one such ligand [1,4].

Angiopep-2 (TFFYGGSRGKRNNFKTEEY, molecular weight 2.4 kDa) is a 19-amino-acid-long oligopeptide that binds to the low-density lipoprotein receptor-related protein-1 (LRP1) [5]. Identified by Demeule and coworkers [6] as part of a family of Kunitz-domain-derived peptides, Angiopep-2 showed greater transcytosis ability and parenchyma accumulation than the protease inhibitor, aprotinin. Aprotinin was used as it possesses a Kunitz protease inhibitor (KPI) domain, which renders it a good substrate for the low-density lipoprotein receptor-related protein (LRP), which facilitates transport across the BBB. 

**Figure 1:**
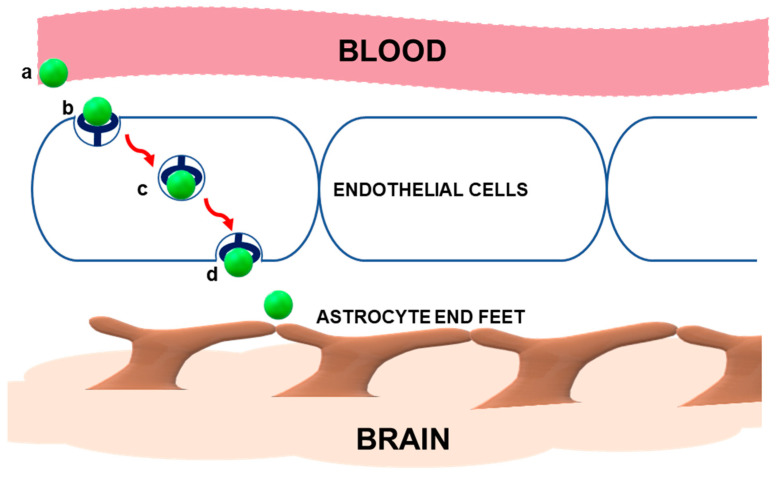
Schematic representation of receptor-mediated transcytosis (RMT) of Angiopep-2. When introduced into the bloodstream, the peptide (a) binds to the low-density lipoprotein receptor-related protein-1 (LRP1) (b) on the apical membrane of brain endothelial cells and initiates invagination of the plasma membrane. The receptor–ligand complex is endocytosed via the intracellular vesicular network (c) and routed to the basolateral membrane, where membrane fusion permits the release of the vesicle contents (d). Angiopep-2 detaches from the receptor and reaches brain cells. Adapted from [15,16].

This study established the framework for the application of Angiopep-2 in brain-directed therapeutics. A representation of RMT of Angiopep-2 is provided in Figure 1. Angiopep-2 has since been appended to anticancer drugs [7,8], a variety of NPs [9,10,11,12,13] and has even been investigated in clinical trials. As a recent example, ANG1005, which consists of three paclitaxel residues linked to Angiopep-2, showed patient benefits in a phase II study of adults with recurrent brain metastases arising from breast cancer [14].

Although drug–ligand conjugates have demonstrated efficacy [17], the association of drugs with BBB ligand-decorated NPs has, in theory, greater benefits. Not only can the drug-loaded nanostructure traverse the BBB but also it has the potential to improve circulation time, encourage cellular uptake, lower the effective dose required, and reduce drug-induced side effects [18]. Interestingly, Angiopep-2-modified nanoparticles were recently shown to enhance transcytosis across the intestinal epithelium with potential for the design of oral delivery systems [19].

In addition to drugs [20], nanotechnology has opened up the possibility of directing other therapeutic agents to the brain. Functional therapeutic gene segments can be introduced via appropriately designed nanoparticles [21,22]. Similarly, small interfering RNA (siRNA), which function in regulating gene expression via RNA-inference (RNAi) can be introduced to inhibit the expression of disease-causing genes in the brain [23,24]. 

**Figure 2:**
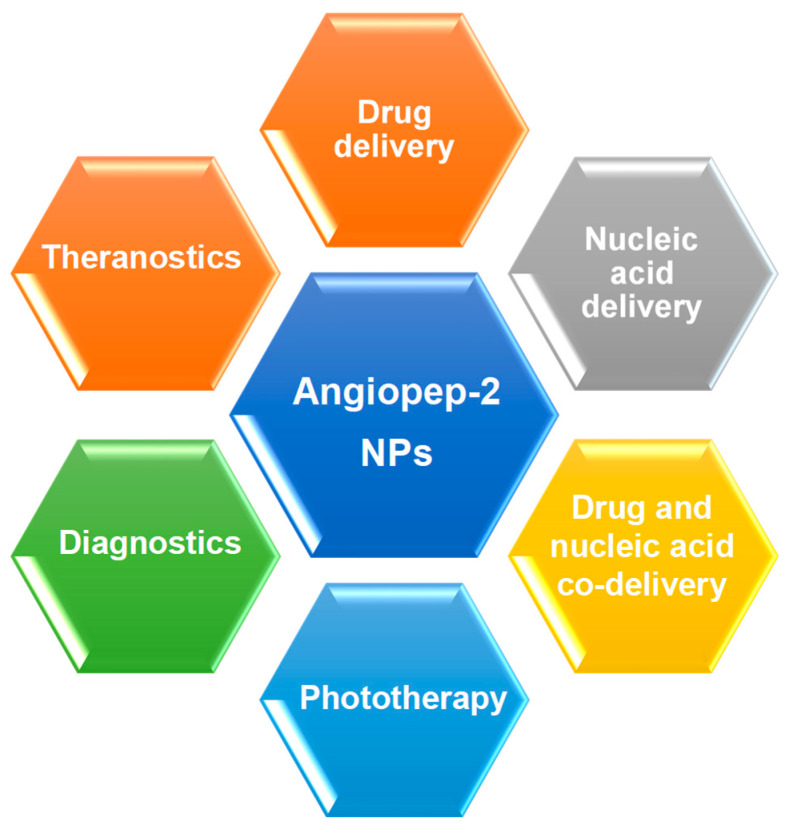
Potential medical usages of Angiopep-2 NPs.

In other instances, micro RNA (miRNA) technology in the form of miRNA mimics and anti-miRNA oligonucleotides (anti-miRs) can be applied to either restore the function of beneficial miRNA or attenuate that of disease-causing miRNA, such as onco-miRs [25]. In the medical field, nanodevices to transport and deliver contrast agents are important diagnostic tools, improving the efficacy of current imaging systems [26]. This review explores the prospects for nanotherapeutics directed towards the brain, which involve Angiopep-2 as a homing device. The major potential applications of Angiopep-2 decorated NPs is broadly outlined in Figure 2.

## 2. Angiopep-2-Decorated Nanoparticles 

**Figure 3:**
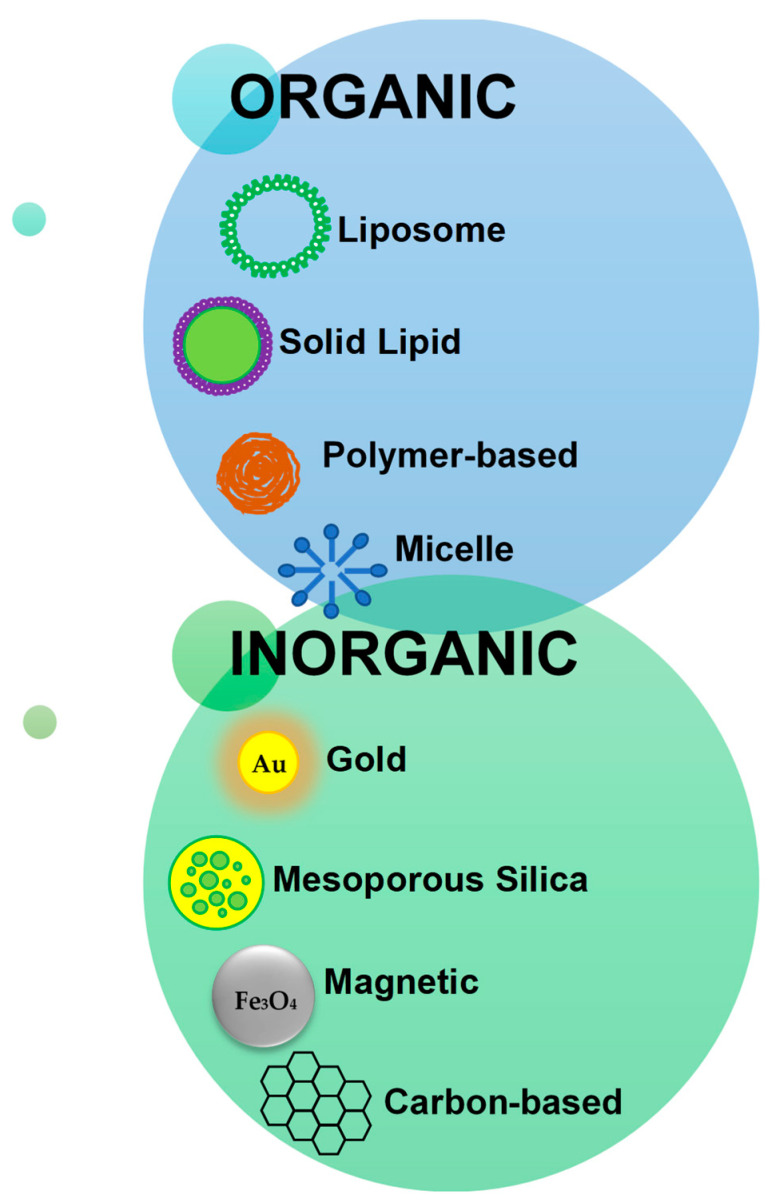
Outline of major nanoparticle classes to which Angiopep-2 has been appended.

Angiopep-2 has been appended to a wide variety of nanostructures for the delivery of therapeutic agents to treat brain disorders, which include cancer, brain injury, stroke, epilepsy, fungal infections, Alzheimer’s disease (AD), and Parkinson’s disease (PD). These encompass both organic and inorganic nanoparticles (Figure 3). 

Liposomes are arguably the most common nano-delivery agents. First described in the 1960s by Bangham and coworkers [27], liposomes are spherical lipid vesicles composed of a phospholipid bilayer that can encapsulate therapeutic agents within the aqueous core. Liposomes are versatile in that they are amenable to several useful modifications, including the appendage of ligands, such as Angiopep-2, on the surface. Danyu and coworkers [28] showed that Angiopep-2 liposomes loaded with the anticancer drug, doxorubicin, had glioma targeting therapeutic effects with reduced toxicity. 

Cationic lipids can be incorporated into liposome formulations to confer a net positive charge that permits convenient electrostatic binding of nucleic acids [29]. Conveniently, cationic liposomes are amenable to carrying both drugs and genes [30]. Angiopep-2-functionalized cationic liposomes were shown to effectively deliver siRNA against Golgi phosphoprotein 3 (GOLPH3) specifically to glioma and inhibit its growth in U87-GFP-Luci-bearing BALB/c mouse models [31]. 

Stealth properties can be conferred using polymer shrouds, such as polyethylene glycol (PEG). Xuan and colleagues [32] demonstrated that the encapsulation of dibenzazepine in PEGylated Angiopep-2-modified liposomes enhanced its cytotoxicity against glioblastoma stem cells. More recently, PEGylated Angiopep-2-modified liposomes were shown to promote the anti-glioma effect of arsenic trioxide [33]. 

An alternative to liposomes is solid lipid NPs (SLNPs). SLNPs are prepared from emulsifier-stabilized lipids that are solid at room temperature [34]. Angiopep-2-grafted SLNPs encapsulating the chemotherapeutic drug, docetaxel showed selective targeting and higher accumulation in the brain than the marketed drug formulation [35].

Angiopep-2 has also been appended to polymer-based NPs. Polymer-based NPs are colloidal systems formulated from natural, synthetic, or semi-synthetic polymers. Polymeric NPs differ in characteristics based on the type of polymer employed. However, they are generally stable in biological fluids and are versatile enough to regulate the stimuli-induced controlled release of their therapeutic payloads [36]. 

Parashar and coworkers [37] recently reported on an Angiopep-2-anchored lipoprotein-coated e-caprolactone nanoparticle to deliver the anti-epilepsy drug, carbamazepine, as an alternative to the standard oral and intravenous routes. Functionalization of a trileucine-stabilized β-poly(l-malic acid) nanoplatform with Angiopep-2 resulted in more effective infiltration of the brain parenchyma than those modified with the brain-shuttle peptide, MiniAp-4, and the transferrin receptor ligands, cTfRL and B6 [38].

Micelles are made up of amphiphilic macromolecules, notably polymers, which form through self-assembly in solution. The hydrophobic segments converge to form a core, while the hydrophilic components form an outer shell. Such micelles are intrinsically stealth NPs capable of evading the reticuloendothelial system [39]. Radiolabeled Angiopep-2-anchored poly (ethylene glycol)-block-poly (d,l-lactide acid) (PEG-PLA) micelles demonstrated high brain accumulation for up to 24 h after intravenous administration in mice [40]. 

Similarly, Angiopep-2-modified PEG-co-poly(ɛ-caprolactone) (PEG-PCL) NPs accumulated at higher levels in the brain cortical layer, lateral ventricle, third ventricles and hippocampus than did unmodified nanoparticles [41]. In the category of polymer NPs, are the dendrimers. Dendrimers are radially symmetrical hyperbranched artificial macromolecules typified by a combination of many functional groups and a compact structure. Surface modification, such as the introduction of the Angiopep-2 moiety, is relatively simple. Moreover, a high level of control can be exerted over dendritic architectures, making them suitable carriers in biomedical applications. 

Polyamidoamine (PAMAM) dendrimers are the most common class of dendrimers utilized to date to deliver nucleic acids [42,43,44,45]. Dendrimers possess an inner alkyl-diamine core and a peripheral shell of tertiary amine branches [46,47]. Angiopep-2-modified PAMAM dendrimers have demonstrated efficacy in delivering doxorubicin to glioma cells [48,49]. Due to the cationic centers of PAMAM at physiological pH, it has also been used for the binding and transport of DNA to the brain in mouse models when associated with Angiopep-2 [50,51].

In recent years, there has been a surge in the investigation of inorganic nanomaterials as carriers of therapeutic agents. These include gold, mesoporous silica, magnetic and carbon-based nanomaterials, and organic/inorganic hybrids. 

Turkevich first reported that the reduction of gold salts in the presence of a reducing agent initiates the nucleation of gold ions [52]. Gold NPs exist in a broad size range of 1 nm to 8 µm and exhibit varying morphology, including nanospheres, nanoclusters, nanorods, nanoshells, nanostars, and nanoprisms [53]. For biomedical applications, the gold core nanostructure is typically modified with an organic monolayer to permit solubility in aqueous environments and control intermolecular interactions of the nanoparticle [54]. 

Key to the tunable characteristics of the monolayer is the appendage of targeting ligands, such as Angiopep-2. In this way, gold nanospheres [55], nanorods [56], and nanoprisms [57] have been directed to the brain in animal models. For example, Angiopep-2-modified hypoxic lipid radiosensitizer-coated gold NPs were shown to enhance the effects of radiation therapy on brain tumor growth in vivo [58].

Another well-studied class of inorganic carriers is the mesoporous silica NP (MSN). MSNs are a specialized form of silica NPs with well-defined porosity and morphology. The porous honeycomb-like structure accounts for a high drug loading capacity and aids in controlled release [59,60]. They are reportedly non-toxic, do not affect healthy tissues, can be imbued with stimuli-responsive features [61], and can be modified to mediate chemo-photodynamic therapy [62]. 

Their behavior in biological systems can be attenuated by controlling the surface chemistry and size [63]. Consequently, in addition to appending Angiopep-2, MSNs directed to the brain have been modified with lipids [64,65] and polymers [66]. In a recent study, Angiopep-2-modified lipid-coated MSNs efficiently loaded paclitaxel, increased glioma cell apoptosis, and prolonged the survival of C6 glioma bearing rats [64]. 

Carbon-based nanomaterials (CBNs) have received extensive attention in biotechnology owing to their tunable surface characteristics and mechanical, electrical, optical, and chemical properties. CBNs, which include graphene oxide, carbon nanotubes, and carbon nanodots, have been functionalized with Angiopep-2. Graphene oxide, as the name suggests, is the oxidized form of graphene, a flat monolayer composed of *sp^2^* hybridized carbon in two-dimensional sheets of a hexagonally arranged honeycomb lattice [67]. 

Graphene is considered superior to other CBNs because it has lower levels of metallic impurities and requires purification processes that are less time-consuming [68]. The modification of graphene oxide with Angiopep-2 increased doxorubicin uptake in U87 MG cells over that of unmodified graphene oxide-doxorubicin and free doxorubicin [69]. Carbon nanotubes (CNTs) are formed by rolling the graphene sheet in a cylindrical structure within a specified nano-diameter [70]. 

PEGylated oxidized multi-walled carbon nanotubes modified with Angiopep-2 demonstrated a combined dual targeting effect in the delivery of doxorubicin to glioma [71]. Carbon nanodots are zero-dimensional spherical allotropes of carbon and are below 10 nm in size. They have great potential for biomedical application due to their biocompatibility, low toxicity, water-solubility, eco-friendly synthesis, conductivity, and desirable optical properties [72]. Angiopep-2 anchored PEGylated carbon nanodots was shown to target C6 glioma cells more effectively than PEGylated carbon nanodots [73].

A relatively recent addition to the growing plethora of inorganic NPs is the superparamagnetic iron oxide NPs (SPIONs). This has been described as one of the most promising tools in theranostic applications. Such NPs typically consist of single or multiple iron oxide cores and are surface modified to promote biocompatibility and stability in biological systems [74]. Hence, brain-targeted magnetic NPs are comprised of hybrid materials. These include Angiopep-2 decorated iron gold alloy NPs [75] and magnetic lipid-polymer hybrid NPs [76]. Of great interest is the possibility of utilizing an external magnetic field to promote deposition of the NP at the desired locality and, in this way, modulating the release of the therapeutics [74]. The advantages and limitations of the major classes of Angiopep-2-modified NPs are summarized in Table 1. 

## 3. Drug Delivery

Most Angiopep-2-modified drug delivery agents have been designed with a view to treat cancers of the central nervous system. Glioblastoma or glioblastoma multiforme (GBM) is the most common and aggressive malignant brain tumor [87]. The current treatment involves a combination of surgery, radiation therapy, and chemotherapy. However, the disease remains highly resistant to treatment [88]. 

The conjugation of Angiopep-2 to NPs is reported to have a dual-targeting effect. Not only does the peptide act as a shuttle to promote transport across the BBB but it is also selective for glioma cells due to the overexpression of LRP1 on their surfaces [55]. Using this concept, several nanosystems have been designed to improve the efficacy of existing chemotherapeutic agents, including doxorubicin, paclitaxel, and docetaxel. Encouragingly, many have demonstrated efficacy in animal models (Table 2). 

For example, treatment of glioma-bearing rats with Angiopep-2 decorated polymersomes loaded with doxorubicin prolonged the survival time compared with unmodified polymersomes and the free drug [89]. In addition, the incorporation of drugs in Angiopep-2 NPs has been linked with the attenuation of side effects. For example, the histopathological analysis of Angiopep-2 decorated nanocarbon tubes carrying doxorubicin suggested lower cardiac toxicity than the free drug.

On the other hand, using a second ligand to bypass the blood-tumor barrier and encourage NP uptake in glioma cells has also been reported. NPs modified with both Angiopep-2 and an activatable cell-penetrating peptide were shown to localize in gliomas with greater efficiency than NPs with a single ligand [90]. Furthermore, docetaxel-loaded Angiopep-2 and TAT functionalized tandem nanomicelles were shown to have a prolonged blood circulation time in mice and inhibited orthotopic U87MG human glioma better than the Angiopep-2 single peptide-functionalized counterpart [91]. 

In the same year, Kim and colleagues [92] demonstrated that the conjugation of both Angiopep-2 and anti-CD133 monoclonal antibody to a liposome was effective in delivering temozolomide to glioblastoma stem cells through the BBB. Dual targeting efficiency was also demonstrated with Angiopep-2 and an AS1411 aptamer covalently linked to a doxorubicin-loaded lipid-capped PLGA NP [93]. 

The incorporation of statins within Angiopep-2-decorated NPs increased LRP-1 expression in brain microvascular endothelial cells and brain metastatic tumor cells. The systemic administration of Angiopep-2-functionalized PEGylated PLGA-PLL NPs co-encapsulating simvastatin and doxorubicin displayed an extended median survival of mice bearing brain metastases due to enhanced BBB transcytosis and the effective targeting of brain metastases [94]. 

Angiopep-2 has also been incorporated into the design of "smart" nanodrugs that are stimuli-responsive to overcome problems that include incomplete drug release or non-site-specific drug deposition. A common strategy involves exploiting unique features of the tumor microenvironment. Ruan and coworkers [55] tethered doxorubicin to an Angiopep-2-modified PEGylated gold NP via a hydrazone bond to permit drug release upon exposure to the acidic tumor locality. More recently, polyacrylic acid was incorporated as part of liposome-silica hybrid nanovesicles to allow the acid-triggered release of arsenic trioxide [95]. 

Recently, the matrix metalloproteinase-1 (MMP1)-rich niche of breast cancer brain metastases (BCBMs) was exploited in the design of NPs that can escape abluminal LRP-1-mediated clearance. PLGA-PLL NPs co-carrying doxorubicin and lapatinib were modified with a MMP-1 sensitive fusion peptide containing HER2-targeting KAAYSL and LRP-1-targeting Angiopep-2. MMP1-triggered cleavage removed Angiopep-2 for augmented accumulation in BCBMs-bearing brains [96]. NPs may also be engineered such that drug release is induced via an externally applied stimulus. Luo and colleagues [97] reported on Angiopep-2-decorated PLGA hybrid NPs that encapsulated an ultrasound contrast agent and doxorubicin. 

High-intensity focused ultrasound (HIFU) was applied to trigger on-demand doxorubicin release at glioblastoma sites resulting in a mean survival time of 56 days for glioblastoma-bearing mice and minimal traces of tumor cells evident in pathological slices. Table 2 summarizes Angiopep-2-decorated nanodrug delivery systems applicable to the treatment of brain disorders. 

## 4. Nucleic Acid Delivery

Gene therapy, the application of nucleic acids to treat disease, promises to revolutionize how brain disorders are addressed. It is theoretically capable of curing the disease rather than merely treating symptoms. Initially envisaged as the introduction of functional gene segments to replace defective genes, gene therapy encompasses more than just the use of therapeutic DNA. Other types of therapeutic nucleic acids applicable to diseases of the brain include small RNA molecules, such as small interfering RNA (siRNA) and micro RNA (miRNA). 

However, using these nucleic acids as medicine necessitates their association with biocompatible carriers to encapsulate, protect, and facilitate cellular entry at the correct site. This synergy created between gene therapy and nanomedicine may be a significant association that can benefit the treatment of various disorders [115]. Angiopep-2 has been appended to various nanostructures for the reliable transport of therapeutic nucleic acids.

DNA-based Angiopep-2-modified NPs have been reported for the treatment of brain cancer and Parkinson’s disease. As an example, Gao and coworkers reported on the delivery of a suicide gene via Angiopep-2 conjugated cationic PEI-PLL-PEG NPs, which penetrated the BBB and accumulated in the striatum and cortex via systemic administration. The system achieved a remarkable anti-tumor effect and survival benefit in an invasive orthotopic human glioblastoma mouse model by inhibiting proliferation and inducing apoptosis [116]. 

Angiopep-2-conjugated dendrigraft poly-L-lysine delivered a therapeutic gene encoding human glial cell line-derived neurotrophic factor in a chronic Parkinsonian model. Pharmacodynamic data revealed that rats in the group with five injections of targeted DNA-bound NPs improved in locomotor activity and apparent recovery of dopaminergic neurons compared to those in other groups [117]. In a proof of principle study, Angiopep-2 and TAT dual modified magnetic lipid-polymer hybrid NPs delivered a reporter gene effectively in C6 cells in a magnetic field [76]. Angiopep-2 NPs have been applied to the delivery of siRNA against genes involved in brain cancer progression and survival. These include GOLPH3, Polo-like kinase 1 (PLK1), vascular endothelial growth factor (VEGF), and vascular endothelial growth factor receptor (VEGFR) genes. 

Zheng and coworkers [118] introduced a polymer capable of stabilizing siRNA by electrostatic hydrogen bonds and hydrophobic interactions. Given that ROS is enriched in cancer cells, the polymer was designed with a ROS-responsive feature to trigger on-site siRNA release. 

With Angiopep-2 functionalization, the polymer successfully delivered siRNA against PLK1 and vascular endothelial growth factor receptor-2 (VEGFR2), leading to effective suppression of tumor growth and significantly improved survival time in mice bearing orthotopic GBM brain tumors. Another Angiopep-2-modified ROS-responsive nanosystem successfully delivered VEGF siRNA into glioma cells. VEGF silencing was accompanied by angiogenesis inhibition and suppressed expression of caveolin-1, which is involved in BBB functional regulation in the occurrence and treatment of glioblastoma [119]. 

Angiopep-2 was also used to functionalize a biomimetic three-layer core-shell nanostructure to deliver siRNA to glioma cells. The nanostructure was designed to release siRNA in the endo/lysosome by charge conversion from negative to positive. This led to highly potent target-gene silencing with a strong anti-GBM effect and minimal side effects [120].

Angiopep-2 has also been involved in miRNA-directed nanotherapy. Liu and coworkers [121] used polymeric NPs to simultaneously supplement the function of miR-124 and inhibit the function of miR-21 to treat glioblastoma. Co-delivery of a miR-124 mimic and anti-miR-21 regulated the mutant RAS/PI3K/PTEN/AKT signaling pathway in tumor cells. This was accompanied by anti-tumor effects, which included reduction of tumor cell proliferation, migration, invasion and angiogenesis, tumor growth suppression, and improved survival time. Table 3 provides an overview of Angiopep-2-functionalised NPs investigated for the delivery of nucleic acids.

## 5. Drug and Nucleic Acid Co-Delivery 

The idea of treating cancer through drug and nucleic acid combination therapy is receiving significant attention. This strategy affords the ability to target more than one mechanism governing the growth and survival of tumors, giving rise to synergistic anticancer effects. Appropriately designed NPs can overcome the challenges associated with the delivery of two therapeutic agents with markedly different physiological properties. 

Nucleic acids are hydrophilic, anionic, high-molecular-weight entities, while the most commonly used chemotherapy drugs are small hydrophobic molecules, thus necessitating different mechanisms for encapsulation [124]. In general, nucleic acids are electrostatically associated with cationic components of the NP, while small molecule drugs are enclosed within them by hydrophobic force, electrostatic interactions, or chemical conjugation [125]. 

Angiopep-2 has served as an essential component of several nanoplatforms for dual agent delivery to the brain. Liposomes, being archetypal delivery systems, have been utilized for multimodal intervention. For example, an Angiopep-2-modified cationic liposome co-carrying the therapeutic gene encoding the human tumor necrosis factor-related apoptosis-inducing ligand (pEGFP-hTRAIL) and paclitaxel was reported to achieve greater apoptosis of glioma cells than single medication systems and the unmodified co-delivery system [126]. 

Liposomes were also dual-functionalized with Angiopep-2 and the tLyP-1 peptide, which targets the neuropilin-1 receptor on glioma cells, for simultaneous anti-angiogenic and apoptotic effects through the delivery of vascular endothelial growth factor (VEGF) siRNA and docetaxel. This system demonstrated superior anti-tumor effects after both intracranial and systemic administration in mice with U87 MG tumors without activating system-associated toxicity or the innate immune response [127]. Sun and colleagues [128] reported on cationic liposomes modified with Angiopep-2 and an aptamer that binds to CD133. 

The co-delivery of survivin siRNA and paclitaxel using this nanocarrier was minimally toxic to brain capillary endothelial cells but selectively caused apoptosis of CD133+ glioma stem cells and improved the differentiation of CD133+ glioma stem cells’ into non-stem-cell lineages. In addition, the system inhibited tumorigenesis, induced CD133+ glioma cell apoptosis, and prolonged survival in intracranial glioma tumor-bearing nude mice. Recently, synergistic tumor-inhibitory effects were also noted with an Angiopep-2 decorated cationic liposome that simultaneously delivered doxorubicin, yes-associated protein (YAP) siRNA, and gold nanorods [129]. 

Wang and colleagues [130] designed Angiopep-2-modified PLGA NPs to encapsulate doxorubicin and siRNA against the EGFR. This co-delivery nanosystem was shown to cause apoptosis of the glioma tissue and prolong lifespan in glioma-bearing mice. Another combinatory anti-glioma system involved the dual release of Gefitinib and GOLPH3 siRNA from an Angiopep-2-modified cationic lipid-PLGA NP. 

This system achieved synergistic anti-EGFR activity in that Gefitinib markedly inhibited EGFR signaling, while GOLPH3 silencing promoted EGFR and p-EGFR degradation [131]. In line with the use of polymers for the design of dual agent nanostructures, Wen and coworkers [132] reported on an Angiopep-2 decorated glycolipid-like co-polymeric micelle for the simultaneous delivery of VEGF siRNA and paclitaxel in vivo. 

Moreover, the nanovector was designed with a redox-responsive feature to trigger the intracellular release of its payload. In the same year, a combination of Temozolomide and PLK1 siRNA by Angiopep-2-modified PEG-PEI-PCL micelles produced enhanced drug efficacy in glioma [133].

## 6. Phototherapy 

In addition to drug and nucleic acid-mediated therapy, brain cancer can be treated via phototherapy. Phototherapy can be subdivided into two main branches, namely, photodynamic and photothermal therapies. Photodynamic therapy (PDT) uses light-sensitive molecules known as photosensitizers, which produce cytotoxic ROS once exposed to a specific wavelength [134]. PDT is minimally invasive because photosensitizers are only cytotoxic when activated in tumor regions. However, PDT cannot treat advanced cancers due to the difficulty of light delivery and the limited penetration depth. 

Photothermal therapy (PTT) is an alternative for advanced tumors, in which photosensitizers absorb near-infrared (NIR) light and release vibrational energy in the form of heat to destroy cancer cells, independent of oxygen [135]. The amalgamation of phototherapy and nanotherapy has led to the construction of NPs that can direct photosensitizers to the tumor site. In other instances, they possess inherent photothermal conversion capability. The latter is true of NPs composed of magnetic and carbon-based materials [136].

Oleic acid-coated upconversion NPs (UCNPs) were conjugated with PEG/Angiopep-2 for the co-delivery of the photothermal agent, IR-780, and photodynamic sensitizer, 5,10,15,20-tetrakis(3-hydroxyphenyl) chlorin (mTHPC) in brain astrocytoma tumors. The photoactivated dual therapies resulted in extensive apoptosis and necrosis of brain tumors, translating into an extended median survival of tumor-bearing mice compared to non-targeted NPs [137]. Recently, Angiopep-2 decorated nanostructured lipid carriers of the photosensitizer, chlorin e6, were evaluated for PDT efficacy in vitro against a glioblastoma model [138].

Phototherapy may also be combined with chemotherapy for synergistic anti-tumor effects. Lu and colleagues [139] developed a multicomponent nanoplatform made up of self-assembled pH-responsive nanodrugs derived from amino acid-conjugated camptothecin and canine dyes coated with an Angiopep-2-conjugated copolymer. The combination of chemotherapy and PTT improved the therapeutic effect with a longer survival time and reduced toxic side effects in orthotopic glioblastoma tumor-bearing nude mice. 

## 7. Diagnostic and Theranostic Applications

Brain cancers are often difficult to detect due to tumors being located deep in brain tissue. More often than not, diagnosis is delayed, which further impedes the success of the treatment administered [140]. Furthermore, accurate tumor imaging is of immense importance in the pre-operative stage and the location of tumor margins [141]. In this regard, Angiopep-2-modified nanosystems have demonstrated great potential. 

Magnetic resonance imaging (MRI) is an imaging technique that uses magnetic fields to assess the morphological structure of organs in the body. It has emerged as a dominant imaging modality in brain cancer diagnosis and clinical staging [142]. However, one of the drawbacks is low sensitivity, which reduces its potential in molecular-level detection. Hence, increasing the contrast between healthy and diseased tissues is of the utmost importance [143]. In this regard, the inherent magnetic properties of iron oxide NPs render them suitable alternatives to conventional contrast agents for MRI [144,145]. 

Chen and colleagues [146] reported on Pluronic® F127-modified water-dispersible poly (acrylic acid)-bound iron oxide NPs modified with Angiopep-2 as brain-directed diagnostic agents. The system demonstrated negligible cytotoxicity, better cellular uptake, and higher *T*2-weighted image enhancement than non-targeted NPs in U87 cells. 

Du and colleagues [147] presented the first report on Angiopep-2 conjugated ultra-small superparamagnetic iron oxide NPs (USPIONs) as *T*1-weighted positive MR contrast agents for intracranial targeted glioblastoma imaging. The nanoprobe showed promise for efficient pre-operative tumor diagnosis and the targeted surgical resection of intracranial glioblastomas. 

Optical imaging using fluorescent NPs is another alternative. Features that include strong signal strength, resistance to photobleaching, tunable fluorescence emissions, and high sensitivity are the impetus for the application of fluorescent NPs in cancer diagnosis [148]. Additionally, fluorescent NPs display stronger fluorescent brightness, better photostability, water dispersibility, and biocompatibility compared with conventional fluorescent dyes. Fluorescent carbonaceous nanodots were prepared from glucose and glutamic acid with long excitation/emission wavelengths to overcome the limitations associated with shorter wavelengths in imaging diseased tissue. 

Decoration with Angiopep-2 resulted in a glioma/normal brain (G/N) ratio of 1.76 [73]. Moreover, the developed system showed good serum stability, hemocompatibility, and low cytotoxicity. Recently, Ren and colleagues [149] designed Angiopep-2-modified Er-based lanthanide NPs with strong NIR IIb fluorescence for imaging-guided surgery of orthotopic glioma. NPs were delivered to gliomas in mice via focused ultrasound sonication to temporarily open the BBB. The highest tumor-to-background ratio (TBR = 12.5) was reported in the targeted NIR IIb fluorescence imaging of small orthotopic glioma through intact skull and scalp was obtained.

Xie and coworkers [141] constructed a MRI/NIR fluorescence dual-modal imaging nanoprobe by combining superparamagnetic iron oxide NPs (SPIONs) with the fluorescent dye indocyanine. This was further modified with the retro-enantiomer of Angiopep-2 to prevent its degradation by enzymes of the blood and cells. In keeping with the idea of dual-modal imaging, Wei and colleagues [150] introduced small-sized iron oxide NPs (SIONs), which were surface modified with Angiopep-2 and the photosensitizer, chlorin e6, to boost fluorescence imaging to support MRI results. 

Angiopep-2 has also served as an essential component of nanosystems that seek to integrate active agents for therapy and diagnosis. Such nanoplatforms, categorized under the broad category of theranostics, promise to significantly benefit the diagnosis, treatment and management of brain cancer. Angiopep-2 was appended to pegylated bubble liposomes at the distal ends of PEG chains. The nanosystem was shown to be capable of encapsulating ultrasound contrast gas and nucleic acids. Systemic administration could serve as a useful device for brain-targeted delivery and ultrasound imaging [151]. 

Crosslinked hyaluronic acid NPs were decorated with Angiopep-2 and formulated to encapsulate gadolinium-diethylenetriamine penta-acetic acid (Gd-DTPA) and the chemotherapeutic agent, irinotecan. The nanosystem showed improved MRI capability, improved uptake in U87 and GS-102 cells, and reduced the irinotecan time response [152]. Lin and colleagues [153] constructed Angiopep-2 coupled bovine serum albumin NPs containing superparamagnetic iron oxide (SPIO), indocyanine green, and the drug, Carmustine. 

The nanoprobes were capable of dual MRI and fluorescence imaging and effective drug delivery. In a deviation from the non-viral NPs discussed thus far, a theranostic NP based on the MS bacteriophage capsid was reported. Angiopep-2 was appended to the external surface, while the interior space was loaded with Mn^2+^ via a porphyrin ring to enable detection via MRI. The inner space can further encapsulate therapeutic agents. Systemic introduction of NPs resulted in dose-dependent, non-toxic accumulation in the midbrain [154].

Iron–gold alloy NPs were also conjugated with Angiopep-2 as a minimally invasive theranostic system. These superparamagnetic NPs enhanced negative Glioma image contrast and exhibited a 12 °C temperature elevation when magnetically stimulated. Angiopep-2 modification resulted in a 1.5-fold higher uptake by glioma cells than fibroblasts, and magnetic field induced hyperthermia decreased cell viability by 90%. Furthermore, treatment resulted in a five-fold decrease in tumor volume and extended survival time in vivo [70]. 

A system integrating targeted brain imaging and chemo- and phototherapy was put forward by Hao and colleagues. PLGA NPs were loaded with indocyanine green as a NIR imaging and phototherapy agent and the anticancer drug, docetaxel. Once modified with Angiopep-2, NIR image-guided chemo-phototherapy resulted in glioma cell death and prolonged survival of glioma xenograft-bearing mice [155]. 

Lipid NPs containing Angiopep-2, a hypoxia-responsive poly(nitroimidazole) 25, indocyanine green, and doxorubicin were proposed for fluorescence-guided surgery chemotherapy, PDT, and PTT combination multitherapy strategies targeting glioma. The study suggested that this nanoplatform may be useful in preventing the post-surgical recurrence of glioma [156].

## 8. Discussion and Conclusions

Tang and colleagues [140] commented that “*BBB-crossing nanotechnology is expected to make a revolutionary impact on conventional brain cancer management*”. In this regard, strategies that exploit Angiopep-2-mediated transport are increasingly important. At present, most Angiopep-2-functionalized nanomedicines have been directed towards the treatment of brain cancers, in particular glioblastoma, which is highly aggressive and responds poorly to the current therapy. 

Advantageously, Angiopep-2 modification has been reported to have BBB and blood-tumor barrier (BTB) dual-penetrating ability. To our knowledge, little comparative data is available with respect to the performance of Angiopep-2 versus other cell-penetrating peptides. Interestingly, enhanced NP functioning has been reported through co-modification with Angiopep-2 and other cell-penetrating peptides. It is worth noting, however, that functionalization of the chemo-therapeutic agent, PAPTP, with either Angiopep-2 or the TAT_48-61_ peptide, permitted similar delivery to the brain in mice [157].

In the past five years, the as-functionalized nanosystems have also demonstrated potential for the delivery of agents to treat other brain disorders, including fungal infections, epilepsy, stroke, brain injury, Parkinson’s disease, and Alzheimer’s disease. For example, gold nanorods functionalized with Angiopep-2 and the D1 peptide that recognizes toxic aggregates of β-amyloid showed efficacy in a *Caenorhabditis elegans* model of Alzheimer’s disease [158]. 

Over the time period considered for this review (2010–2021), Angiopep-2-decorated nanostructures have been employed to deliver a vast array of therapeutic and diagnostic agents. These include chemical compounds; the nucleic acids DNA, siRNA, and miRNA; photosensitizers; and contrast agents. In addition, Angiopep-2-modified nanoparticles are applicable to immunotherapy. Wang and colleagues [159] reported that Angiopep-2 and IP10-EGFRvIIIscFv fusion protein-modified NPs can recruit activated CD8+ T lymphocytes to glioblastoma cells. 

While the application of Angiopep-2-modified NPs in phototherapy is well documented, another novel physical method of destroying brain cancer cells, sonodynamic therapy (SDT), based on ultrasound stimulation, has been reported. Qu and colleagues [160] designed an innovative “all-in-one” nanosensitizer platform by combining the sonoactive chlorin e6 and an autophagy inhibitor, hydroxychloroquine, in Angiopep-2-modified liposomes to simultaneously induce apoptosis and inhibit mitophagy in glioma cells. 

As in the aforementioned study, Angiopep-2 NPs, are amenable to the integration of dual- and multimodal therapy. Angiopep-2 nanosystems can also be engineered to behave in a stimuli-responsive fashion to permit a controlled and sustained release of their therapeutic cargo. Moreover, their potential in theranostics has been highlighted in recent years. Encouragingly, there is a growing body of in vivo data to support the further design of multifunctional Angiopep-2-modified nanomedicines. Overall, there is a great need to translate the in vitro and in vivo achievements of BBB-crossing nanotherapeutics to the clinic. 

In addition to the impact of NP shape, size, and charge on BBB-transcytosis, the number of Angiopep-2 residues displayed on the surface may have a significant influence. The multimeric association between Angiopep-2 peptides and the LRP1 was shown to increase the intracerebral uptake of NPs significantly [161]. The local flow environment is also a necessary consideration for in vitro modelling of the performance of NPs functionalized with Angiopep-2. Studies with Angiopep-2-labelled liposomes suggested that blood flow can influence the binding and BBB penetration of NPs [162].

In summary, this review highlighted the role of Angiopep-2-modified NPs in the diagnosis and treatment of brain disorders. With greater streamlining of NP design, advances in BBB modelling and further in vivo testing, it is envisaged that Angiopep-2-based nanosystems may make their way into the clinic for the routine assessment and treatment of brain cancer and other disorders of the brain in the years to come. 

## Figures and Tables

**Table 1:** Advantages and disadvantages of some Angiopep-2-modified nanoparticles.

Nanoparticle	Advantages	Disadvantages	References
Liposomes 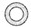	Biocompatible and easy to prepare. Can deliver anionic and cationic material. Tunable composition. Can be modified for cell-specific targeting.	High production cost Short shelf-life Possibility of drug leakage	[77]
Polymeric Nanoparticles 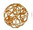	Biocompatible and biodegradable. Long half-life. High drug stability.	Complicated synthesis methods. Purification is difficult. Difficult to produce on a large-scale.	[78]
Micelles 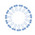	Biocompatible. Can potentiate controlled drug release. Tunable chemical and physical properties.	Low drug loading capacity, which is dependent on micelle concentration	[79]
Dendrimers 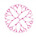	High drug loading capacity. Chemistry can be easily modified. Ability to penetrate biological barriers. Can be modified for cell-specific targeting.	Some dendrimers can be cytotoxic.	[80]
Gold 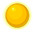	Biocompatible. Small size with high surface area to volume ratio. Surface can be easily modified. Potential for green synthesis.	Potential toxicity if retained in the body over a long period.	[53,81]
Mesoporous silica 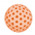	Biocompatible and biodegradable. High surface area with large pore sizes. Has well-defined surface properties.	Preparation can be complex. Varied size distributions can occur.	[59]
Carbon-based Nanoparticles 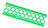	Can be functionalized. Potential for photothermal therapy (graphene oxide and carbon nanotubes). Carbon nanodots have potential for imaging.	Toxic if not functionalized.	[82,83,84,85]
Magnetic Nanoparticles 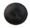	Biocompatible and easy to synthesize. Surface can be modified. Magnetic properties can be exploited for targeting and controlled release of therapeutic.	Has no internal loading capacity.	[86]

**Table 2:** Angiopep-2-modified nanoparticles that have been used for drug delivery.

Nanoparticle	Drug/s	Disorder Treated	Test System	Reference
Liposome-silica hybrid	Arsenic trioxide	Glioma	C6 glioma-bearing rats	[95]
PAMAM dendrimer	Doxorubicin	Glioma	C6 glioma cells	[48]
poly(dimethylsiloxane)-poly(2-methyloxazoline) (PDMS-PMOXA) diblock copolymer	Doxorubicin	Glioblastoma	U87MG glioblastoma cells	[98]
Carboxymethyl chitosan nanogel	Doxorubicin	Glioblastoma	-	[99]
lipid-poly-(metronidazoles) hypoxic radiosensitized- polyprodrug	Doxorubicin	Glioma	C6 glioma cells Glioma-bearing ICR mice	[100]
lipid-poly (hypoxic radiosensitized polyprodrug)	temozolomide	Glioblastoma	C6 glioma cells Glioma-bearing ICR mice	[101]
PEG-*b*-poly(ε-caprolactone) (PEG-*b*-PCL)	Doxorubicin	Primary CNS lymphoma	SU-DHL-2-LUC lymphoma xenograft mice model	[20]
PEG-*co*- poly(ε-caprolactone) polymersome	Doxorubicin	Glioma	C6 glioma cells C6 glioma-bearing rats	[89]
PCL-PEG	Ginsenoside-Rg3	Glioma	C6 glioma cells	[102]
PEGylated gold	Doxorubicin	Glioma	C6 glioma cells C6 glioma-bearing mice	[55]
Poly (lactic-co-glycolic acid) (PLGA)-based mesoporous silica	Doxorubicin Paclitaxel	Glioma	Human brain micro- vascular endothelial cells BBB model	[66]
PEGylated PLGA-PLL	Doxorubicin Simvastatin	Brain metastases	-	[94]
Biomimetic nanoparticles	Doxorubicin Lexiscan	Glioblastoma	U87MG human glioblastoma tumor- bearing nude mice	[103]
Graphene oxide	Doxorubicin	Glioma	U87 MG cells/ mouse xenograft	[69]
PEGylated oxidized multi-walled carbon nanotubes	Doxorubicin	Glioma	C6 glioma cells C6 glioma bearing mice	[71]
HIFU-responsive PLGA hybrid	Doxorubicin	Glioblastoma	Glioblastoma-bearing mice	
PLGA Gold	Docetaxel	Glioma	-	[104]
Solid lipid nanoparticles	Docetaxel	Glioblastoma	U87MG glioblastoma cells GL261 mouse glioma Glioblastoma- induced C57BL/6 mouse model	[35]
PEG-PCL	Paclitaxel	Glioma	3D glioma tumor spheroids Intracranial glioma mouse model	[105]
Lipid-coated mesoporous silica nanoparticles	Paclitaxel	Glioma	C6 glioma cells C6 glioma-bearing rats	[64]
Phospholipid-functionalized mesoporous silica	Paclitaxel	Glioma	HBMEC cells C6 glioma cells	[65]
PEGylated poly propyleneimine (PPI) dendrimers	Paclitaxel	Glioblastoma	C6 glioma cells Co-culture BCECs model	[106]
redox-responsive virus- mimicking polymersome	Saporin	Glioblastoma	U-87 MG glioblastoma cells U-87 MG human- glioblastoma mouse model	[107]
PEG-PE polymeric micelles	Amphotericin B	Meningo- encephalitis	Immunosuppressive murine *Cryptococcus* *neooformans* meningo- encephalitis model	[108]
PE-PEG polymeric micelle	Amphotericin B	CNS fungal infections	-	[109]
Ceria	Edaravone	Ischemic stroke	-	[110]
PEG-PLGA	Tanshinone IIA	Ischemic stroke	-	[111]
PEG-PAMAM nanoparticle	Scutellarin	Ischemic stroke	-	[112]
Electro-responsive hydrogel	Phenytoin sodium	Epilepsy	Amygdala kindling seizure model	[113]
Lipoprotein-coated e-caprolactone	Carbamazepine	Epilepsy	Adult male albino rats	[37]
PEGylated 2-methoxy estradiol micelle	2-Methoxy estradiol	Cerebral ischemia- reperfusion injury	PC12 cells	[114]

**Table 3:** Angiopep-2-modified nanoparticles for nucleic acid delivery.

Nanoparticle	Nucleic Acid	Nucleic Acid Details	Disease	Test System	Reference
PAMAM-PEG	DNA	pORF-TRAIL	Glioma	C6 glioma cells	[51]
PAMAM-PEG	DNA	pEGFP-N2	-	BCEC Balb/c mice	[50]
PEI-PLL-PEG	DNA	Herpes simplex virus type I TK gene	Glioblastoma multiforme	Human GBM mouse model	[116]
dendrigraft PLL	DNA	Gene encoding human glial cell line-derived neurotrophic factor	Parkinson’s	Rotenone-induced chronic model of Parkinson’s disease	[117]
Cationic liposome	siRNA	GOLPH3 siRNA	Glioma	U87-GFP-Luc-bearing BALB/c mouse models	[31]
Polymeric	siRNA	siPLK1 siVEGFR2	Glioblastoma	GBM brain tumor mouse model	[118]
Polyplex	siRNA	-	Glioma	Glioma mouse model	[122]
Chimeric polymersomes	siRNA	siPLK1	Glioblastoma	U-87 MG cells Glioblastoma mouse model	[123]
Biomimetic nanoparticles	siRNA	-	Glioblastoma	U87MG- Luc human glioblastoma mouse model	[120]
ROS cleavable thioketal-linked glycolipid-like nanocarriers	siRNA	siVEGF	Glioblastoma	U87 MG cells	[119]
Polymeric	miRNA	miR-124 anti-miR-21	Glioblastoma	U87MG-Luc human glioblastoma tumor mouse model	[121]

## Data Availability

Not applicable.
